# Eculizumab as a Rescue Therapy in Prolonged Myasthenic Crisis in the Intensive Care Unit: A Case Series

**DOI:** 10.1007/s12028-025-02237-w

**Published:** 2025-03-20

**Authors:** Hacer Durmus, Arman Çakar, Yesim Gülşen Parman

**Affiliations:** Department of Neurology, Istanbul Faculty of Medicine, Istanbul University, Istanbul, Turkey

Myasthenia gravis (MG) is a rare autoimmune disorder affecting the neuromuscular junction caused by antibodies targeting postsynaptic membrane proteins [1]. In anti-acetylcholine receptor antibody–positive (AChR+) generalized MG (gMG), terminal complement activation leads to AChR antibody–mediated destruction of the motor endplate, disrupting neuromuscular transmission. Myasthenic crises are potentially life-threatening [2] and represent the most severe presentation of MG, characterized by profound muscle weakness, bulbar symptoms, and potential for respiratory failure. Intravenous immunoglobulin (IVIG) and plasma exchange (PLEX) are conventional treatments for myasthenic exacerbations [2]. Eculizumab (ECU) is a humanized monoclonal antibody that binds to complement protein C5 and inhibits the activation of terminal complement, thereby protecting the neuromuscular junction from these destructive effects. The open-label extension phase of REGAIN, along with several observational studies, has supported favorable outcomes and safety with ECU in refractory gMG, but its role in severe myasthenic exacerbations or crises remains to be fully elucidated [3].

## Case Series

Three female patients with AChR + gMG who were intubated due to myasthenic crises (Myasthenia Gravis Foundation of America clinical classification class V) and unresponsive to conventional rescue therapies in the intensive care unit (ICU) for at least 1 month before receiving ECU were included in the study. Following informed written patient consent, data were retrospectively collected from medical records. Patients received an induction dose of 900 mg on day 1 and at weeks 1, 2 and 3, followed by a maintenance dose of 1200 mg at week 4 and every 2 weeks thereafter. Patients were vaccinated against *Neisseria meningitidis* serogroup A, C, W, Y (Nimenrix), and B (Bexsero, administered twice with a 1-month interval) before initiation of ECU. Vaccination was tolerated well without exacerbation of myasthenic symptoms. All patients received prophylactic antibiotics until 2 weeks after vaccination (penicillin V 500 mg twice a day or amoxicilline 500 three times a day). None of the patients had active infections at the time of ECU initiation. Patient 1 was receiving meropenem for nosocomial pneumonia, which continued for 10 more days after post-ECU initiation before transitioning to penicillin V 500 mg twice a day for prophylaxis.

The mean age at the time of myasthenic crisis was 51.3 years (range 31–62 years). Demographic and clinical characteristics of the patients at baseline are summarized in Table 1. The oldest patient was newly diagnosed with MG, presenting with acute severe bulbar symptoms and respiratory distress necessitating ICU admission. The second patient had long-standing, well-controlled gMG but experienced an exacerbation following major surgery (aortic valve replacement due to aortic stenosis) complicated by nosocomial pneumonia and sepsis in the ICU. Although responsive to antibiotics, multiple ventilator-weaning attempts failed, leading to the diagnosis of myasthenic respiratory weakness. The third patient, with recurrent thymoma, presented with worsening myasthenic symptoms likely triggered by a viral infection. An arterial blood gas analysis indicated respiratory failure, which was initially managed with noninvasive ventilation before ICU transfer for mechanical ventilation the next day.

**Table 1:** Basic clinical features and treatments prior to ECU in the ICU

Case ID	Sex	MG subtype	Age at MG crises (yr)	Cause of crisis	Disease duration (mo)	Thymus pathology	Mechanical duration prior to ECU (day)	Mechanical duration after to ECU (day)	Treatments during the crisis before ECU
1	F	LOMG	73	First manifestation of MG	New diagnosis	No thymoma	53	12	IVIG 2 g/kg over 5 days and a month later 0.4 g/kg. Prednisolone 1 mg/kg daily
2	F	TMG	61	Major surgery	72	Thymoma	62	10	IVIG 2 g/kg over 5 days and a month later 0.4 g/kg. Prednisolone 1 mg/kg daily
3	F	TMG	37	Viral infection	2	Recurrent thymoma	33	8	IVIG 2 g/kg over 5 days and a month later 0.4 g/kg. Prednisolone 1 mg/kg daily

ECU, eculizumab, F, female, ICU, intensive care unit, IVIG, intravenous immunoglobulin, LOMG, late-onset MG, MG, myasthenia gravis, TMG, thymoma-associated MG

**Fig. 1:**
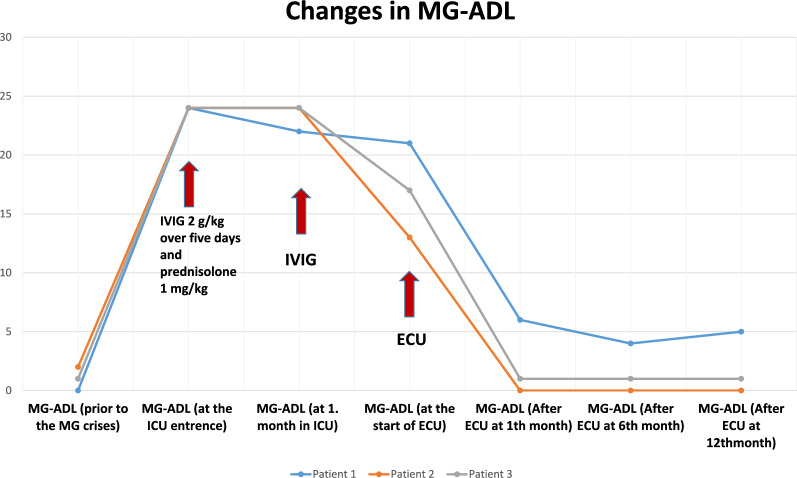
Change in mean Myasthenia Gravis Activities of Daily Living (MG-ADL) total score during treatment with eculizumab (ECU). ICU, intensive care unit, IVIG, intravenous immunoglobulin

All patients received IVIG (2 g/kg over 5 days) upon presentation and were maintained on high-dose oral corticosteroids (1 mg/kg prednisolone) for at least 1 month. A second course of IVIG was preferred over PLEX because of sepsis or hemodynamic instability. All had severe bulbar symptoms and extremity weakness. Because of the lack of a clinically meaningful response to conventional rescue therapy and prolonged ICU stay, ECU was administered. The mean ICU duration before ECU initiation was 49.3 days (range 33–62 days), with all patients requiring tracheostomy due to prolonged intubation. All patients were successfully extubated and discharged from the ICU within 2 weeks of starting ECU, with no significant adverse effects reported. The mean Myasthenia Gravis Activities of Daily Living (MG-ADL) score before ECU treatment was 17 (range 13–21), decreasing to 2.3 (range 0–6) after 1 month and further to 2 (range 0–5) after 12 months (Fig. 1).

Myasthenic crises are rare but life-threatening complications of MG, requiring timely and effective management. ECU has demonstrated efficacy in reducing disease burden in refractory gMG, but its role in acute myasthenic crises remains under investigation. Previous case reports suggest that ECU may facilitate weaning from mechanical ventilation, particularly in patients unresponsive to conventional therapies [4–9]. Our case series provides additional real-world evidence supporting ECU as a potential rescue therapy for patients with myasthenic crisis in ICU settings. The rapid and sustained improvement in MG-ADL scores, along with successful extubation and ICU discharge, supports its potential to alter disease trajectory in patients experiencing prolonged myasthenic crises.

However, further studies are necessary to determine the optimal timing of the complement inhibitors and to address additional safety considerations related to vaccination and prophylactic antibiotic use.

Emerging options to manage gMG in clinical practice may ultimately lead to alterations in therapeutic algorithms. Several case series have supported the rapid effect and safety of another novel therapeutic option, neonatal Fc receptor (FcRn) antagonists, in myasthenic crisis. There is no direct comparison of complement inhibitors and FcRn antagonists for myasthenic crisis. The safety profile of FcRn antagonists may favor their use in patients with ongoing infections in the ICU, where complement blockade and vaccination pose additional risks. Both therapies are expensive, but cost-effectiveness may vary based on regional health care policies. FcRn antagonists are not approved in our region.

Given the life-threatening nature of myasthenic crises and effectiveness of complement inhibitors as well as FcRn antagonists in achieving rapid clinical improvement in gMG, further investigations are warranted to explore full therapeutic potential of these newer treatments in this critical condition.

## Data Availability

All analyzed data are presented in the article and are available on reasonable request from qualified investigators.
